# Role of Ubiquitination in IGF-1 Receptor Signaling and Degradation

**DOI:** 10.1371/journal.pone.0000340

**Published:** 2007-04-04

**Authors:** Bita Sehat, Sandra Andersson, Radu Vasilcanu, Leonard Girnita, Olle Larsson

**Affiliations:** Department of Oncology and Pathology, Cancer Centre Karolinska (CCK), Karolinska Institutet and Karolinska University Hospital-Solna, Stockholm, Sweden; Ordway Research Institute, Inc., United States of America

## Abstract

**Background:**

The insulin-like growth factor 1 receptor (IGF-1R) plays numerous crucial roles in cancer biology. The majority of knowledge on IGF-1R signaling is concerned with its role in the activation of the canonical phosphatidyl inositol-3 kinase (PI3K)/Akt and MAPK/ERK pathways. However, the role of IGF-1R ubiquitination in modulating IGF-1R function is an area of current research. In light of this we sought to determine the relationship between IGF-1R phosphorylation, ubiquitination, and modulation of growth signals.

**Methodology:**

Wild type and mutant constructs of IGF-1R were transfected into IGF-1R null fibroblasts. IGF-1R autophosphorylation and ubiquitination were determined by immunoprecipitation and western blotting. IGF-1R degradation and stability was determined by cyclohexamide-chase assay in combination with lysosome and proteasome inhibitors.

**Principal Findings:**

IGF-1R autophosphorylation was found to be an absolute requirement for receptor ubiquitination. Deletion of C-terminal domain had minimal effect on IGF-1 induced receptor autophosphorylation, however, ubiquitination and ERK activation were completely abolished. Cells expressing kinase impaired IGF-1R, exhibited both receptor ubiquitination and ERK phosphorylation, however failed to activate Akt. While IGF-1R mutants with impaired PI3K/Akt signaling were degraded mainly by the proteasomes, the C-terminal truncated one was exclusively degraded through the lysosomal pathway.

**Conclusions:**

Our data suggest important roles of ubiquitination in mediating IGF-1R signaling and degradation. Ubiquitination of IGF-1R requires receptor tyrosine kinase activity, but is not involved in Akt activation. In addition we show that the C-terminal domain of IGF-1R is a necessary requisite for ubiquitination and ERK phosphorylation as well as for proteasomal degradation of the receptor.

## Introduction

In the past few years, the insulin-like growth factor 1 receptor (IGF-1R) has emerged as a receptor tyrosine kinase (RTK) with important roles in cancer biology. The physiological responses to IGF-1R tyrosine kinase activation are diverse and include differentiation, proliferation, protection from apoptosis, cellular transformation, and cancer progression [1]–[3]

The IGF-1R is a tetrameric receptor tyrosine kinase consisting of two ligand-binding extracellular α-subunits and two β-subunits composing a transmembrane domain, an intracellular tyrosine kinase domain and a C-terminal domain [4]. Ligand-receptor interaction results in phosphorylation of tyrosine residues in the tyrosine kinase (TK) domain (spanning from amino acid 973–1229) of the β-subunit. The crystal structure of the inactive and phosphorylated kinase domain has provided a molecular model of the IGF-1R catalytic activity [5]. In unstimulated state, the activation loop, containing the critical tyrosine (Y) residues 1131, 1135 and 1136, behaves as a pseudosubstrate that blocks the active site. Upon ligand binding the three tyrosines of the activation loop are transphosphorylated by the dimeric subunit partner. Phosphorylation of Y1135 and Y1131 destabilizes the auto-inhibitory conformation of the activation loop, whereas phosphorylation of Y1136 stabilizes the catalytically optimized conformation [5], allowing substrate and ATP access. The phosphorylated tyrosine residues serve as docking sites for other signaling molecules such as insulin receptor substrate 1–4 (IRS-1-4) and Shc, leading to the subsequent activation of the phosphatidyl inositol-3 kinase (PI3K), the mitogen-activated protein kinase (MAPK), and the 14-3-3 pathways [1], [4], [6], [7].

Recent data has shown that IGF-1R is a substrate for ubiquitination, however, the role is unclear[8]–[11].Two E3 ligases, Mdm2 [8] and Nedd 4 [9], have been demonstrated to be involved in mediating the covalent attachment of ubiquitin moieties to lysine residues in IGF-1R. In Mdm2-mediated ubiquitination, β-arrestin function as a molecular scaffold in bridging the ligase to the receptor [12]. Similarly, Nedd4-mediated IGF-1R ubiquitination requires Grb10 to function as an adapter protein [9]. However, in spite of identification of these ligases involved, the understanding of the functional consequences and target residues are still limited.

In general, activated receptors must be cleared from the cell surface in order to desensitize the cell to mitogenic signals [13]–[15], and numerous studies have suggested a role for ligand-induced receptor internalization in the consequent degradation/desensitization of activated receptors [16]. There are several endocytic pathways that can mediate internalization of cell surface receptors, some of which are dependent on receptor ubiquitination [17], [18]. The final step of receptor life cycle is degradation, which occurs either in lysosomes or in proteasomes or in both. Degradation through the proteasomal pathway requires that the receptor has undergone ubiquitination, however ubiquitinated receptors can also be degraded by lysosomes.

The fact that IGF-1R is ubiquitinated makes it as a possible substrate for proteasomal degradation. However, several studies have demonstrated that degradation of epidermal growth factor receptor (EGFR), being the most investigated RTK in this respect, is mediated by lysosomal proteases [13], [19]–[21]. The pathway through which IGF-1R is degraded is still an issue of debate. Evidence for involvement of the proteasomal pathway are mainly based on observations that the degradation of the IGF-1R can be blocked by the proteasome inhibitor MG132 [9], [22]. However, it has recently been revealed that MG132 is not a specific inhibitor of proteasomal proteases and may also block the lysosomal pathway [23], [24]. Accordingly, the degradation pathway responsible for downregulation of IGF-1R is still unclear.

Using different mutated constructs we aimed to identify functional sites and domains of IGF-1R necessary for receptor ubiquitination and to address whether ubiquitination is involved in control of signaling and degradation.

## Results

### Functional sites and domains of IGF-1R

**Figure 1:**
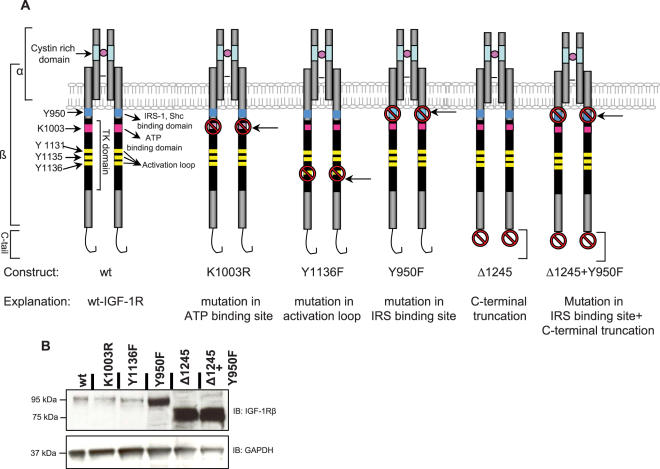
Wild type and mutant IGF-1R constructs. A. IGF-1R is a heterodimeric receptor with two identical ß-subunit and two identical α-subunits. The α-subunit is completely extracellular, while the ß-subunit spans the membrane. The intracellular portion (residues 931–1337) has a TK domain with intrinsic kinase activity. Important tyrosine residues in the TK domain, know as activation loop, are (Y1131, Y1135 and Y1136) shown in green. Tyrosine residue Y950 (in blue) is, when phosphorylated, a docking site for binding of signaling molecules. Other important regions of the receptor are the ATP binding site (K1003) and the C-terminal domain important for phosphorylation and signaling, respectively. B. The expression of all IGF-1R variants were investigated on cells growing under basal conditions (complete medium supplemented with serum). Cell lysates were subjected to western blotting using antibodies to IGF-1R (β-subunit) and GAPDH (loading control).

The β-subunit of the IGF-I receptor spans the membrane and contains the TK domain, responsible for the overall kinase activity of the receptor (Fig. 1A). The lysine K1003 serves as ATP-binding site and an IGF-1R construct with a point mutation at this site (K1003R) cannot be autophosphorylated [25]. The tyrosine Y1136 is located in the activation loop and is important for stabilization of kinase activity [5]. An IGF-1R construct with a point mutation at this site (Y1136F) exhibits a decreased kinase activity [26]. A phosphorylated Y950 is important for binding of IRS-1 and Shc, and therefore is necessary for normal signaling (Fig. 1A). Consequently, an IGF-1R construct with a single mutation at Y950 site (Y950F) leads to impaired signaling [27]. The C-terminal domain is also involved in signaling [28], [29]. A C-tail truncated IGF-IR (Δ1245, missing the last 92 amino acids) (Fig. 1A) exhibits impaired signaling [28], [29], whereas the Y950F+Δ1245 construct beside lacking the C-terminal domain also has impaired IRS-1/Shc binding (Fig. 1A).

In order to study these constructs in cell systems we used R- cells (IGF-1R knockout) stably transfected with wild type IGF-1R (here referred to as wt), Y1136F, Δ1245 and Y950F+Δ1245 as well as transiently transfected with K1003R. All constructs are of human origin.

The expression level of the above IGF-1R variants under basal conditions is demonstrated in Fig. 1B. As shown, Δ1245 and Y950F+Δ1245 cells exhibit the strongest receptor expression.

### Phosphorylation is necessary for ubiquitination of IGF-1R

**Figure 2:**
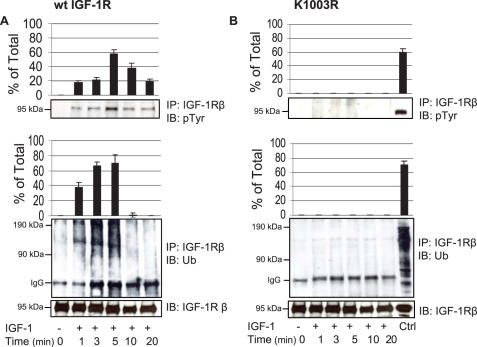
Ubiquitination of IGF-1R is phosphorylation dependent. After 24-h serum starvation wt IGF-1R (A) and K1003R (B) cells were stimulated for indicated time points with 50 ng/ml IGF-1. Lysates were immunoprecipitated (IP) with anti-IGF-1Rß (H60), and blotted with either anti-phosphotyrosine (pY99) or anti-ubiquitin (p4D1). The graphs represent quantification (% of loading control) of three independent experiments. Means and SDs are shown. Equal loading was confirmed by stripping and re-immunoblotting with anti-IGF-1Rß (C20).

Upon ligand (IGF-1) stimulation the IGF-1R is rapidly autophosphorylated and, as recently demonstrated, ubiquitinated [8], [9]. However, the relationship between these two modifications has not been studied in detail. To investigate this issue, wt IGF-1R and K1003R cells were serum depleted for 24 h and then stimulated with IGF-1 for the indicated times (Fig. 2). IGF-1R was immunoprecipitated from cell lysates and analyzed by western blotting for phosphotyrosine and ubiquitin modifications. Phosphorylation of wild type IGF-1R was detectable 1 min after ligand exposure, peaking at 5 min but still detectable at later time points (10 and 20 min) (Fig. 2A). The ubiquitination of the receptor which starts simultaneously as phosphorylation, is visualized as a high-molecular smear>90 kDa, peaking at 5 min (Fig 2A). The graphs show the quantified signals of receptor phosphorylation and ubiquitination based on 3 separate experiments. In the kinase inactive IGF-1R cells (K1003R) where the receptor phosphorylation is abolished no ubiquitination was observed, indicating that IGF-1R kinase activity is required for IGF-1-induced ubiquitination.

### C-terminal domain is required for IGF-1R ubiquitination

**Figure 3:**
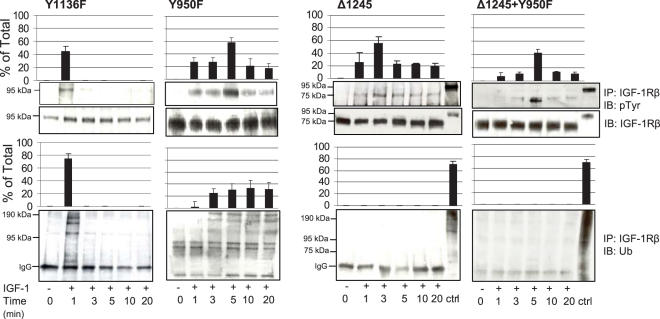
C-terminal domain is essential for ubiquitination of IGF-1R. After 24-h serum starvation Y1136, Y950F, Δ1245 and Y950F-Δ1245 cells were stimulated for the indicated time points with IGF-1. Determinations of IGF-1R phosphorylation and ubiquitination were performed as described in Fig. 2. The investigated signals from three independent experiments were quantified (% of loading control) and presented in graphs above each blot. Means and SDs are shown.

Next we sought to investigate the potential requirement of specific sites/domains of the IGF-1R for receptor ubiquitination. In this respect, we used cells stably transfected with the 4 aforementioned IGF-1R mutants (Fig. 1). As shown in Fig. 3, Y1136F cells (with impaired IGF-1R kinase activity due to a point mutation at Y1136 site in the activation loop) is phosphorylated and ubiquitinated shortly after ligand stimulation, however the phosphorylation and ubiquitination patterns are transient when compared with wild type (Fig. 2A). Both phosphorylation and ubiquitination of the receptor were detectable exclusively after 1 min exposure to IGF-1, however not after longer stimulation times (3–20 min). Cells expressing Y950F receptors (with impaired signaling due to reduced binding of Shc and IRS-1 to the receptor) displayed phosphorylation and ubiquitination with similar kinetics as the wild type IGF-1R (cf. Fig. 2A), although the ubiquitination signals persisted longer in Y950F cells (Fig. 3). Interestingly, whereas the C-tail truncated IGF-1Rs in Δ1245 and Y950F-Δ1245 cells are fully phosphorylated (compared to the wild type receptor), the ubiquitination capacity is completely abolished (Fig. 3). This suggests that the C-terminal domain is critical for IGF-1 mediated ubiquitination.

### IGF-1R ubiquitination is important for ERK activation

**Figure 4:**
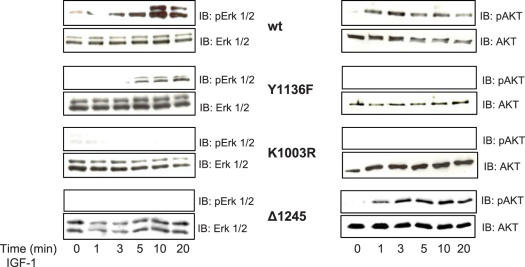
Ubiquitination of IGF-1R is crucial for ERK signaling. After 24 h serum starvation wt, Y1136F, K1003R, and Δ1245 cells were stimulated with IGF-1 for the indicated times and lyzed. The lysates were resolved by SDS-PAGE and analyzed by western blotting with anti-phospho-Akt, anti-phospho-ERK. The blots were then stripped and reprobed with anti-Akt, anti-Erk antibodies to demonstrate equal loading. The experiments were repeated with similar results.

Since we now have identified an IGF-1R construct with deficient ubiquitination capacity we sought to investigate whether ubiquitination influences the signaling responses mediated by IGF-1R. We stimulated serum starved wt, K1003R, Y1136F and Δ1245 cells with IGF-1 for different time points (0–20 min). Phosphorylation of ERK1/2 was assessed as a measure of MAPK activation and phosphorylation of Akt as a measure of PI3K activation. The levels of expression of phospho-ERK1/2 and phospho-(S473) Akt were investigated by immunoblot analysis (Fig. 4). Akt phosphorylation induced by wild type IGF-1R was seen after 1 min of IGF-1 stimulation, peaked at 3 min and was maintained until the final time point. IGF-1 induced phosphorylation of ERK1/2 became detectable at 3 min and reached a maximum at 10 min after which the level declined (Fig. 4). As expected K1003R cells showed neither Akt nor ERK phosphorylation. Consistent with previous report [30] Y1136F transfected cells did not exhibit any Akt activation, whereas ERK1/2 activation resembled that of wild type IGF-1R (Fig. 4). Intriguingly, IGF-1 stimulation of C-terminal truncated IGF-1R in Δ1245 cells activated Akt but not ERKs. This suggests that IGF-1R ubiquitination, shown for wt and Y1136F in Fig. 2 and 3, is important for signaling of the MAPK pathway. Consequently, the transient ubiquitination (detectable only at 1 min of ligand stimulation) observed for the Y1136F mutant seems to be sufficient to activate ERKs. On the other hand, it is evident that activation of the PI3K/Akt signaling pathway requires a higher IGF-1R kinase activity.

### Ubiquitination status correlates with pathway of IGF-1R degradation

We investigated degradation of the different IGF-1R constructs using cyclohexamide (CHX) to block *de novo* protein synthesis as described elsewhere [31]–[33]. Cells, grown under basal conditions, were exposed to CHX for 6 or 12 h. To find out whether the receptors were degraded by lysosomes and/or proteasomes, lysosomal and proteasomal inhibitors were utilized. Cloroquine is a lysosomotropic weak base, which diffuses across membranes in a concentration-dependent manner. It rapidly becomes protonated thereby neutralizing the acidic environment of endocytic vesicles [34]. Whereas, epoxomicin specifically targets the proteasomes by inhibiting primarily the chymotrypsin-like activity [24]. In contrast to peptide aldehyde proteasome inhibitors like MG132, epoxomicin does not inhibit non-proteasomal proteases such as trypsin, chymotrypsin, papain, calpain, and cathepsin B at concentrations of up to 50 µM [24]. In addition, epoxomicin is a more potent inhibitor of the chymotrypsin-like activity than lactacystin and the peptide vinyl sulfone NLVS [24].

**Figure 5:**
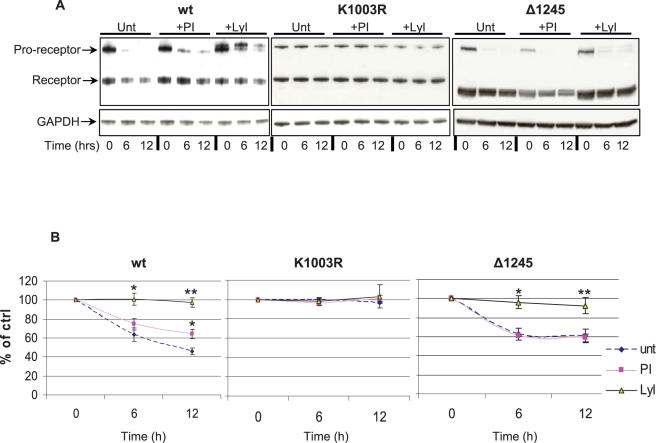
IGF-1R is degraded through both proteasomes and lysosomes. A. Wt, K1003R, and Δ1245 were either untreated (Unt) or pre-incubated with epoxomicin (PI) or chloroquine (LyI) as described in Materials and methods. Cycloheximide (CHX) (50µg/ml) was added and the cells were further incubated for 6 or 12 h. The cells were lyzed and subjected to SDS-PAGE and western blotting with antibody to IGF-1Rß. The proreceptor and 95 kDa β-subunit of IGF-1R is indicated. B shows quantified data of IGF-1R, as normalized with GAPDH. Means and SDs of three separate experiments are shown. * *P,<0.05*; **P,<0.005.

Wt, K1003R and Δ1245 cells were pre-treated with epoxomicin or chloroquine before treatment with CHX and the levels of IGF-1R expression were analyzed by western blotting (Fig. 5A) and quantified by densitometry (Fig. 5B). As seen in Fig 5, in the absence of inhibitor wild type IGF-1R was reduced by approximately 50% after 12 h. The lysosome inhibitor (LyI) completely prevented the degradation, while the proteasome inhibitor (PI) had a moderate, although statistically significant (*P<0.05*), stabilizing effect on IGF-1R. Fig. 5 also shows that the ATPM receptor, being deficient in both ubiquitination and phosphorylation, is not degraded at all during the 6 or 12 h experiments. These data suggest that phosphorylation is necessary for degradation and moreover indicate that the lysosomes represent the main pathway by which the wild type IGF-1R is degraded. Nevertheless, the fact that IGF-1R degradation is somewhat delayed (approximately with 20%) in the presence of PI (Fig. 5B) indicates that the proteasomal activity facilitates IGF-1R degradation. We tried to address whether this effect is directly due to involvement of proteasomes in IGF-1R degradation or is mediated through other mechanisms. It has been suggested for some receptors (e.g. interleukin 2 receptor and EGFR) that ubiquitination is important for endosomal sorting where ubiquitin seems to be required to prevent internalized receptors from recycling by shunting them into a pathway that results in lysosomal degradation [35]–[37]. Another explanation could be that the free pool of ubiquitin dramatically decreases after proteasome inhibitor treatment leading to altered ubiquitination patterns of the receptor, followed by altered internalization and lysosomal degradation. Therefore, we investigated degradation of IGF-1R in Δ1245 cells (C-terminal truncated IGF-1R), which is phosphorylated but defective in ubiquitination. The C-terminal truncated IGF-1R is entirely protected from degradation by the lysosome inhibitor, whereas proteasome inhibitor had no protecting effect on it (Fig. 5B). Taken together, this suggests that the delay in degradation of wild type IGF-1R caused by proteasome inhibition might be a direct effect of receptor ubiquitination and not due to indirect effects such as altered internalization caused by lack of free ubiquitin

**Figure 6:**
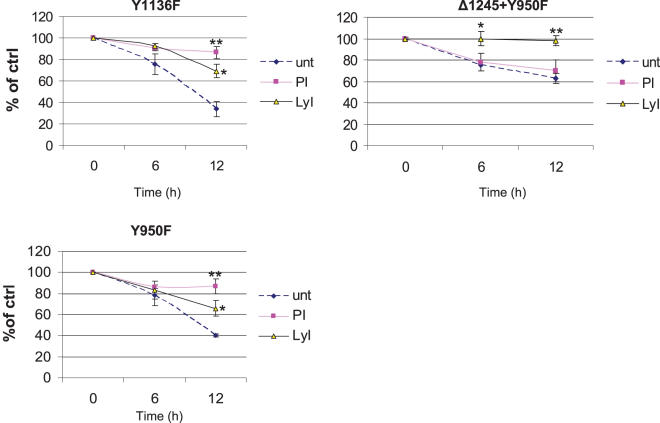
Intact Y1136 and Y950 are important for lysosomal degradation of IGF-1R. Y1136F, Y959F, and Y950F-Δ1245 cells were pre-incubated without or with PI or LyI and treated with CHX as described in Fig. 5. After western blotting quantification of IGF-1R, as normalized with GAPDH, was performed. Means and SDs of three different experiments are shown. *P,<0.05; ** *P,<0.005*.

Unexpectedly, we observed that the mutant receptors in Y1136F and Y950F cells, which both exhibited IGF-1R ubiquitination (Fig. 3), are mainly degraded through proteasomes (Fig. 6). Untreated Y1136F is degraded by 70% after 12 h. Lysosome inhibitor decreased this degradation by 40% while the proteasome inhibitor abolished it totally. The same pattern was seen in Y950F cells. In Y950F-Δ1245 cells, with truncated C-tail and impaired IRS-1/Shc binding, the receptor showed essentially similar responses as in Δ1245 cells (Fig. 5 and 6).

## Discussion

In this study we sought to shed light on regulation of and role of IGF-1R ubiquitination. The results presented here show that phosphorylation of the receptor is necessary for its ubiquitination. Furthermore, we can state that the C-terminal domain of the receptor is needed in this context. In wild type IGF-1R the profile of ubiquitination matches its phosphorylation status. However, C-tail truncated IGF-1R cannot be ubiquitinated in spite of its normal kinase activity. Since, C-terminal truncation of IGF-1R inhibits receptor ubiquitination, one could speculate that the lysine(s) required for ubiquitination are located in the C-tail. However, out of the 29 lysine residues in the β-subunit of the IGF-1R only three are in the C-tail domain. Although these three lysines might function as ubiquitin acceptors, another possibility is that the C-terminal domain functions as an E3 ligase binding site. Further studies are, however, required to understand the distinct mechanism by which C-terminal truncation inhibits ubiquitination of IGF-1R. Importantly, when ubiquitination of IGF-1R is prevented by C-tail truncation the receptor is degraded explicitly through the lysosomes, suggesting a role of IGF-1 induced ubiquitination in proteasomal degradation of the receptor.

Intriguingly, we observed that the IGF-1R mutant Y1136F is mainly degraded via the proteasomes. Since this mutant did not exhibit any Akt phosphorylation, but intact phosphorylation of ERK1/2, it is possible that the PI3K/Akt pathway has a positive regulatory influence on lysosomal degradation. A similar predominance of proteasomal degradation occurred in case of the Y950F mutant, which has an impaired IRS-1 binding [38], [39]. Further support for a role of the PI3K/Akt signaling pathway in this context is contributed by our observations that all IGF-1R constructs exhibiting fully detectable Akt phosphorylation are mainly degraded by the lysosomes. Interestingly, accumulating data have emphasized a role of IGF-1 activated Akt in phosphorylation and nuclear localization of Mdm2 [40]–[42]. These results could offer an explanation regarding the different pattern of IGF-1R degradation in the various mutants. Consistently, in mutant cell lines with impaired Akt activation (Y950F and Y1136F), Mdm2 is probably not phosphorylated with an increased cytoplasmic pool of Mdm2 as a consequence. Since Mdm2 is a ligase required for ubiquitination of IGF-1R [8], the raised levels of cytoplasmic Mdm2 would increase ubiquitination of IGF-1R followed by proteasomal degradation.

In summery we have identified a receptor domain critical for the IGF-1R ubiquitination and show that this post-modification is dependant of phosphorylation of the receptor. Furthermore, we show the co-existence of both proteasomal and lysosomal pathway for IGF-1R degradation, in which PI3K/Akt signaling may be important for the lysosomal pathway and ubiquitination for the proteasomal one. Also, IGF-1R activated MAPK/ERK signaling may be controlled by ubiquitination of the receptor.

## Materials and Methods

### Reagents

Polyclonal IGF-1R antibodies (C-20, and H-60), monoclonal antibody to phosphotyrosine (PY99) and monoclonal antibody to ubiquitin (p4D1) were purchased from Santa Cruz Biotechnology Inc., Santa Cruz, CA. Anti-pErk1/2, anti-Erk1/2, anti-pAkt (serin 473), and anti-Akt antibodies were purchased from Cell Signaling Technology, Danvers, MA. Protein G Sepharose from Amersham Pharmacia Biotech (Uppsala, Sweden). All other reagents unless stated otherwise were from Sigma (St Louis, MO, USA).

### Cell cultures

Cell lines used are derived from 3T3-like fibroblasts isolated from mouse embryos with a targeted disruption of the IGF-IR gene (R-cells) [43]. The cells R- cells were stably transfected with human wild type IGF-1R or mutated variants of human IGF-1R (Y950F, Δ1245, Y950F-Δ1245 and Y1136). These cell lines were kind gifts from Dr. Renato Baserga (Thomas Jefferson University, Philadelphia, PA). R- cells were transiently transfected with a plasmid containing IGF-1R with mutation at the ATP binding site (K1003R). This plasmid was provided by R Baserga. Cells transfected with wild type IGF-1R and K1003R were maintained in Dulbecco's modified Eagle's medium (DMEM) supplemented with 10% (v/v) fetal calf serum, 10 mM L-Glu, 5 mg/ml penicillin/streptomycin and G418 (Promega). Cells transfected with Y950F-Δ1245, Y950F and Y1136F were routinely cultured in DMEM supplemented with 10% fetal bovine serum (FBS) 10 mM L-Glu, 5 mg/ml penicillin/streptomycin and hygromycin (250 µg/ml). Cells transfected with Y950F were routinely cultured in DMEM supplement with 10% FBS, 10 mM L-Glu, 5 mg/ml penicillin/streptomycin and puromycin (2 µg/ml).

### Transient transfection

R- cells were seeded to 90% confluence in 10-cm dishes (Falcon) with plasmids using Lipofectamine 2000 (Life Technologies, Inc., Grand Island, NY), according to the manufacturer's instructions. 24 h after transfection cells were splited into 6 well plates and cultured for an additional 24 h in the presence of 0.6 mg/ml G418. During the last 24 h, cells were starved and then stimulated for indicated times with 50 ng/ml IGF-1. Cells were lyzed in lysis buffer (0.5% Triton-x-100, 0.5% Doc Deoxycholic acid,150 mM NaCl, 20 mM Tris pH 7.5, 10 mM EDTA, 30 mM sodium pyrophosphate, 10% glycerol, 1 mM phenylmethylsulfonyl fluoride, protease inhibitor cocktail tablet (Roche, Mannheim, Germany), phosphatase inhibitor 1 and 2, and 10 mM N-Ethylmaleimide). Protein extracts were prepared for immunoprecipitation or western blot analyses.

### SDS/PAGE and western blot analysis

Cell lysate were distracted as described above. Protein samples were dissolved in a sample buffer containing 0.0625 M Tris·HCl (pH 6.8), 20% glycerol, 2% SDS, bromophenol blue, and 2β-mercaptoetanol. Samples corresponding to 50–100 µg of cell protein were separated by 7.5% or 4–12% gradient sodium dodecyl sulphate polyacrylamide gel electrophoresis (SDS PAGE). Molecular weight markers (Bio-Rad) were run simultaneously. After SDS/PAGE the proteins were transferred onto nitrocellulose membranes (Hybond, Amersham, UK) and blotted with the indicated antibodies. This was followed by washes and incubation with a HRP conjugated secondary antibody (ImmunoPure antibody, Pierce), and detected with (Hyperfilm-ECL, Amersham, UK).

### Immunoprecipitation

Cells were cultured to subconfluency in 6-cm plates. The cells were serum-depleted for 24 h, and then stimulated by IGF-1 (50 ng/ml) at indicated time points. For determination of IGF-1R phosphorylation and ubiquitination, cell lysates were extracted with lysis buffer as described above. Twenty µl of protein G Sepharose and 1 µg of antibody were added to 1 mg of protein material. After overnight incubation at 4°C on a rocker platform, the immunoprecipitates were collected by centrifugation in a microcentrifuge at 2,500 rpm for 2 min. The supernatant was discarded, whereupon the pellet was washed 2 times with lysis buffer and 1 time with PBS and then dissolved in a sample buffer for SDS/PAGE and further was analyzed by western blotting.

### Degradation assay

Protein degradation was assessed by cycloheximide-chase assay as to previously described [31], [32]. The effect of lysosome inhibitor (LyI), cloroquine and proteasome inhibitor (PI), epoxomicin on the stability of the entire IGF-1R pool was examined by immunoblot analysis at 6- and 12 h after treatment with cycloheximide. Cell lines with wild type and different IGF-1R mutants were grown on plates and the levels of IGF-1R were studied with and without PI and LyI. The experiment was preformed in complete culture medium in order to follow receptor downregulation under physiological conditions. The protein synthesis of the cells was subsequently inhibited with cycloheximide (CHX, 50 µg/ml) that was maintained during the whole experiment. In cases where proteasomes were inhibited, epoxomicin was added to a final concentration of 100 nM, 6 h [24], [44] prior to addition of cycloheximide. Lysosomes were inhibited by adding chloroquine to a final concentration of 50 µM 30 min [45], [46] before addition of cycloheximide and were present throughout the experiment.
