# Expression of Concern: Role of Ubiquitination in IGF-1 Receptor Signaling and Degradation

**DOI:** 10.1371/journal.pone.0319195

**Published:** 2025-02-06

**Authors:** The PLOS One Editors

After this article [1] was published, concerns were raised about Figs 2–4.

Specifically:

- In Fig 2, the wt IGF-1R IB: IGF-1Rβ panel appears similar to lanes 1–6 of the K1003R IB: IGF-1Rβ panel.
- In Fig 3, there appear to be discontinuities in the background of the Δ1245 IB: IGF-1Rꞵ panel
- In Fig 4, the following panels have no detectable background signal:
  ○ Y1136F IB: pAKT
  ○ K1003R IB: pAKT
  ○ Δ1245 IB: pERK 1/2

The authors’ response did not resolve these concerns. The authors stated that the underlying data are no longer available. In the absence of the raw data, the issues with these figures cannot be resolved.

In light of the above concerns, the *PLOS One* Editors issue this Expression of Concern to inform readers that findings in these figures should be interpreted with caution.
